# “It seems like a never-ending job”: voices of female caregivers of older adults in the rural communities

**DOI:** 10.3389/fpubh.2025.1751524

**Published:** 2026-01-15

**Authors:** Nasreen Lalani, Bhagyashree Katare, Evans Appiah Osei, Siqi Yang, Sampada Wagle, Julian L. Gallegos, Abidemi Mary Ajuwon

**Affiliations:** Purdue University, West Lafayette, IN, United States

**Keywords:** selfcare, caregivers, burden, palliative, wellbeing

## Abstract

**Background:**

Rural female caregivers of older adults face significant caregiving challenges that puts them at high risk for poor *self-care* and wellbeing. Limited studies have examined the self-care needs of caregivers from a gender equity and social perspective.

**Objective:**

Our study aims to explore the self-care needs and preferences of rural female caregivers and underlying key processes contributing toward their health and well-being.

**Methods:**

A qualitative descriptive design was used for the study. A purposive sample of (*n* = 20) rural female caregivers was obtained. In-depth individual interviews were conducted for data collection. Each interview was about 45-60 min in duration. Thematic analysis approach was used to analyze the data.

**Findings:**

Major themes identified included: no time for personal care, feelings of guilt and helplessness, giving up on career goals and aspirations, marital devotion, and lack of social services and support. Common self-care strategies reported were use of social media, online church meetings and meditation, participation in voluntary services, and owning a pet.

**Conclusion:**

Rural female caregivers need well informed and evidence-based respite policies and programs to support their overall coping, resilience, and well-being. Caregiving policies need to consider gender inclusive, faith and value-based wellness programs in rural communities. Technology can also offer innovative solutions to engage caregivers and promote their self-care.

## Introduction

*Caregiving roles* combined with rurality bring several stressors and challenges (1–3). In the United States, the number of informal or family caregivers (FCgs) is rising from 43.5 million in 2015 to 53 million in 2020 (4, 5). One third of the caregivers are females, among which 80% are caring for someone aged 50 years or older (6). They provide 24.4 h of care on an average/week and have been reported to spend 50% more-time caregiving than their male counterparts; even when both partners work full-time (7). Over half (53.8%) of these women are aged 65 or older, have multiple chronic illnesses, limited education, and little or no health insurance (8, 9). For some, caregiving turns to be a 24/7 job due to the unpredictability of the care recipient’s behavior (10–12). They not only provide medical or symptom support but are also involved in the social, emotional, and spiritual care of their older family members. The immense caregiving burden often prevents FCgs from meeting their own self-care needs, placing them at high risk for poor coping, diminished health, and reduced well-being (13–16). Moreover, the consequences of caregiving negatively affect the caregiver’s health, leading to a decline in cognitive and physical well-being, impairing the informal caregiver’s overall health, and impacting their performance throughout the day, which in turn affects the person with a dementia diagnosis (17).

About two-thirds of rural caregivers experience social isolation, lack of appetite, and sleep disorders, worse physical health, anxiety, and depression (9–11, 18, 19). Prior research consistently shows that caregivers tend to prioritize the needs of older adults over their own self-care and wellbeing (20, 21). The structural and systemic barriers, gender roles, burden of employment, household responsibilities, and role expectations, all such factors combined reduces caregivers’ ability and time to self-care (15, 22, 23). However, limited studies have examined how caregiving in *rural areas* affects self-care and well-being from gender and equity perspectives. Not addressing these concerns can further heighten care disparities and impair the quality of life of rural female caregivers.

## Definitions and conceptual clarifications

For the purposes of this study, we have defined rural or rural areas using the US Census Bureau and Rural–Urban Commuting Area (RUCA) codes as any area with a population of <50,000 people (24). FCgs are defined as a person who provides unpaid care to a family member or friend who needs assistance with daily tasks due to aging, illness, or disability (25). In addition, self-care is defined as individual practice such as healthy eating, sleeping, balancing solitude and social interaction, stress relief, and spiritual connectedness to keep physical, psychosocial, and spiritual health and well-being (21).

## Conceptual framework

Our study was guided by the socio-ecological resilience framework and social role theory. The socio-ecological resilience framework asserts that one’s ability to navigate resources and sustain self-care and well-being is influenced by the underlying social and cultural processes such as personal agency, family relationships, available social support systems, religion, culture, and environment (26–28). Similarly, social role theory explains how societal and gender roles and expectations shape individuals’ behaviors and identities and influence their division of labor, roles and relationships and overall quality of life (29, 30). Rural women carry multiple caregiving roles, juggle several tasks, and thus neglect their self-care and well-being. The theories explain how rural women navigate complex caregiving roles within resource-limited environments and how these roles are shaped by broader societal and cultural norms that prioritize family care often at the expense of the caregivers’ personal health and wellbeing.

## Objective

Given above, our study aims to explore self-care needs and preferences of rural female FCgs and determine the underlying key processes contributing toward poor coping, resilience and well-being of rural female FCgs of older adults.

### Study setting

The study was conducted in the rural counties of Indiana, US. Indiana is situated in the Midwestern US and has a total population of 6.87 million in 2023. Indiana is divided into 92 counties, 42 of which are considered rural, with a total population of approximately 900,000 residents. A rural county is defined as one with a population of fewer than 40,000 residents and a population density of less than 100 people per square mile (31–33). Rural areas have a higher proportion of older adults with multiple chronic illnesses, such as heart disease, cancer, and dementia. Limited access to palliative care services in these areas consequently increases caregiving burden and adversely affects caregivers’ self-care and well-being (4).

### Study design

The design for this study was a qualitative descriptive design.

### Female FCg recruitment and sample

To explore and document the perspectives of rural female FCgs of older family members, we conducted in-depth qualitative interviews to gather data about their self-care needs and preferences and to explain underlying mechanisms associated with their coping and wellbeing. A purposive sample of 20 participants was recruited. Inclusion criteria as any female 18 years or older, spouse, parent or sibling caring for an older family member with any chronic illness or disability at homes, long-term care facility or any other residential care facility. Female FCg recruitment was done in collaboration with the Federally Qualified Rural Health Clinics, Rural Extension, rural hospitals, and clinics located in different rural counties of Indiana. Recruitment flyers were distributed across rural clinics, hospitals, churches, community recreation centers, libraries, local businesses, and through social media and community newsletters. In addition, research assistants conducted in-person recruitment at rural fairs and other community events.

### Data collection

A semi-structured interview guide was developed based on existing caregiving and palliative care literature and refined through consultation with research experts, rural extension educators, and healthcare providers to ensure relevance to rural caregiving contexts. The finalized interview guide is provided in Appendix A.

Data were collected between December 2023 and May 2024. Following recruitment and eligibility confirmation, the Principal Investigator (PI; N. L.) and a graduate research assistant (GRA; S. Y.) contacted participants via email to schedule interviews at their preferred date, time, and mode (in-person, telephone, or videoconferencing).

Interviews were conducted privately either in person, by telephone, or via Zoom or Microsoft Teams, depending on participant preference. The initial 12 interviews were done by PI and GRA whereas the remaining eight interviews were completed by GRA only. Of the 20 interviews conducted, 2 were in person, 4 by telephone, and 14 via videoconferencing platforms. Interviews explored participants’ caregiving roles and expectations, self-care experiences, needs and preferences, and coping strategies. At the conclusion of each interview, participants rated their caregiving-related stress using a subjective scale ranging from 0 (no stress) to 10 (highest stress). Interviews lasted approximately 45–60 min. No follow-up interviews were conducted.

The sample size was determined based on data saturation, with data collection concluding when no new information emerged. All the interviews were recorded and transcribed by the PI (N. L) and two GRAs (E. A. O and S. Y). Repetition of responses emerged by the 17th female FCg. Three additional interviews were conducted to confirm that no new information was arising, and responses remained consistent with previously collected data. Data collection was therefore concluded at 20 female FCgs.

## Ethical considerations

IRB approval was obtained (Purdue University IRB #2020–150). Verbal consent was obtained prior to interviews, and demographic data were collected. Personal identifiers were removed, and all data were stored securely in an encrypted institutional repository. Participants received a $25 e-gift card for their contribution.

## Data analysis

All interviews were transcribed and analyzed manually by the research team using a thematic analysis approach. This was done by the Principal Investigator (PI), two graduate research assistants (GRAs), and three undergraduate research assistants. The team conducted a thematic analysis, guided explicitly by the Socio-Ecological Resilience Theory and Social Role Theory as sensitizing frameworks (34). These theories informed how the researchers critically read, interpreted, and organized the data within the broader ecological and social-role contexts relevant to rural family caregiving.

A preliminary codebook was developed after several close readings of the transcripts. Data were analyzed manually and classified into initial codes and categories to create conceptual linkages and to illuminate underlying meanings. Codes and categories were generated iteratively, reviewed, and finalized by consensus to ensure rigor and credibility. The PI and the two GRAs independently coded all transcripts. Weekly analytic meetings were held to compare interpretations, discuss theoretical fit with the frameworks, identify discrepancies, and refine the codebook. During these meetings, the team explicitly examined how emerging codes reflected multi-level resilience processes (individual, interpersonal, community, societal) and core social-role constructs (role expectations, socialization, gender roles, division of labor, and role conflict/strain).

For example, the raw data excerpt: *“Sometimes I had to run out in the morning to provide care. I do not have time to grab anything to eat—just a cup of coffee, which I was not used to. I feel like I’m suffering from memory problems too because I’m constantly around so much repetition…”* was initially coded as *fatigue, lack of appetite,* and *mental exhaustion*. These codes were then organized into the higher-order category “Lack of time for basic physical care.” Within the theoretical analysis, this category was understood as reflecting individual-level strain and interpersonal caregiving demands (Socio-Ecological Resilience Theory) as well as role expectations and role strain (Social Role Theory), given the pressure to prioritize caregiving tasks over personal well-being.

Through iterative refinement, codes were grouped into higher-level categories and ultimately synthesized into a thematic structure. This thematic list was reviewed and finalized after multidisciplinary team discussions to ensure rigor, credibility, and strong theoretical grounding. One of the final themes, “No Time for Personal Care,” emerged as a clear example of how caregiving responsibilities intersect with ecological constraints and socially constructed role expectations in rural environments.

Reflective notes and team discussions were used to enhance trustworthiness. The study adhered to the Consolidated Criteria for Reporting Qualitative Research (COREQ) and the Standards for Qualitative Research (SRQR) to ensure comprehensive transparency and methodological rigor (35, 36).

## Findings

### Demographic profile

Table 1 provides a detailed summary of the female FCgs’ demographic profile. We found the following themes under the study: No time for personal care, feelings of guilt and helplessness, giving up on career goals and aspirations, marital devotion, and lack of social services and support. Female FCgs reported their *self-care* coping strategies as use of social media, voluntary services, and owning a pet. Findings from Stress Rating Scale showed about two-thirds (70%) of female FCgs rated their stress levels at 8, indicating increased stress levels reported on a scale of 0 to 10 (with 10 being the highest).

**Table 1:** Socio-demographic profile.

Demographic variables	Frequency/percentages *N* = 20
Age	
Mean	57.7 years
40–69	11 (55%)
50+	9 (45%)
Marital status	
Married	14 (70%)
Unmarried	6 (30%)
Caregiver-patient relationship	
Caring for Parent/ In-laws	17 (85%)
Spouse	2 (2%)
Sibling	1 (5%)
Average income levels	
60,0000–70, 000	10 (50%)
Below 60,000	10 (50%)
Care giving duration	
0–2 years	9 (45%)
6 + years	11 (55%)
Caregiving hours	
0-20 h/h/week	11 (55%)
More than 20 h/week	10 (45%)
Diagnosis of older adults	
Dementia	7 (35%)
COPD/Heart failure	4 (15%)
Liver failure	1 (5%)
Parkinson	2 (10%)
Cancer	2 (5%)
Multiple chronic illness such as dementia, heart failure, and diabetes	4 (20%)
Caregiver Stress Rating on a scale of (0–10) (0 = no stress, 10 = highest stress)	
8 and above	14 (70%)
4–7	4 (20%)
Below 4	2 (10%)

### No time for personal care (individual/interpersonal/role strain/conflict)

Most female FCgs shared that their caregiving roles and responsibilities leave little or no time for themselves. Female FCgs reported a lack of rest, sleep, and appetite. Most of them prioritized the safety and comfort of their older adults over their own health and well-being. Female FCgs shared that their diet and appetite patterns have suffered, they lack time for proper cooking and rarely have time to eat a balanced or a full meal. One of them said as *“sometimes I had to run out in the morning to provide care. I do not have time to grab anything to eat, just a cup of coffee which I was not used to”.* (FCgs-9, 50 years).

Female FCgs also reported feelings of anger, frustration, anxiety and signs of forgetfulness or memory loss due to over exhaustion. “*I feel like I’m suffering from memory problems too because I’m constantly around so much repetition. She will sometimes ask me the same questions six times in a minute, and because of this, I feel like I cannot think as clearly*.” (FCgs- 1, 44 years).

A 44-year-old female caregiver caring for her mother with dementia and her teenage sister with a neurological disorder voiced out that “*caregiving is mentally and emotionally taxing, leaving her no time for her self-care.”* Reflecting on her experience, she expressed feelings of exhaustion and expressed as *“it seems like a never-ending job*” and *“will she ever be out of it.?.”* Female FCgs reported that caregiving demands a lot of patience and courage. A female FCg caring for her spouse with dementia reported herself as getting emotionally unstable and experiencing feelings of frustration, anger, and anxiety with time. She added as:

*“….for the last five years or so, I felt like my life was just trying not to make him upset. Very draining though…sometimes when I was in the shower, I would hear him Call My Name and as soon as I would get out, I would wrap the towel and come out cause it’s a little way from the living room and he would say, I forgot where you were.” (FCgs-2, 72 years).*

### Feelings of guilt and helplessness (individual/interpersonal/role strain/conflict)

Female FCgs experienced guilt for not being able to control their emotions and getting angry or irritable toward their older adults sometimes resulting from the extended hours of caregiving, irregular dietary and sleep patterns and constant pressure of caregiving demands and expectations. Caregivers reported that sometimes they had to stay late at the assisted living or long-term care facility or are called in the middle of the night to respond to emergencies. This constant pressure sometimes left them feeling overwhelmed as though they were on the verge of giving up or losing themselves. One of them said: *“So, I feel like I’m. I’m just sort of losing my mind. I feel like I’m losing myself, I feel bad about myself and have no control over the situation….” (FCgs-2, 72 years).* Another female FCg caring for her father with diabetes and dementia described her guilt as:

*“As a caregiver, one of the biggest struggles I face is my own character. When I am stressed, I often become irritable, and I really do not want to be that way. When I notice myself getting irritable, I do not like it. This is one of the major emotional challenges I deal with in caregiving, and it’s my quick irritation that bothers me the most.” (FCgs- 13, 58 years).*

For caregivers, it was hard to watch their loved ones dying, they often found themselves helpless and constantly grieving over the loss of their loved ones. “*It’s hard to see your mom like slowly dying in front of your face every day.”* (FCgs 3, 55 years). For some, it was difficult to talk about their loss and letting out their feelings in front of other people due to their cultural reasons. They expressed that *they feel stuck and hopeless, and like they ‘ll never get out of those feelings*. Some also expressed guilt over not being able to present for their loved ones all the time due to their other responsibilities (Table 2).

**Table 2:** Themes, subthemes/codes and categories.

Themes	Subthemes	Codes/categories	Socio-ecological resilience/social role theory mapping
Burdens of rural family caregiving	No time for personal care Feelings of guilt and helplessness Giving up on career goals and aspirations Marital devotion Lack of social services and support	Fatigue, lack of sleep and appetite, mental exhaustion, multiple tasks to finish, Managing multiple caregiving and personal responsibilities Anger, role conflict, helplessness, hopelessness, guilt for not being able to provide enough care, and grief. Not working, turn down employment, Love, sacrifice, devotion, commitment Lack of paid caregivers, unaffordable care, lack of access, long distances	a. Individual level/Interpersonal level/role expectations/strain b. Individual level /Interpersonal level/Role conflict/strain c. Individual level /Societal/policy level/Role expectations/Division of labor d. Individual level /Interpersonal level/Gender roles/Socialization/Role expectation e. Community, Societal and Policy Level /Interpersonal level/Division of labor/Role strain
Caregiver coping and self-care strategies	Use of social media Online church meetings and meditation apps Participation in voluntary services Owning a pet	Emails, sending text messages, Facebook Virtual church meetings, meditation apps, loneliness Gardening, walking, mindfulness, exercise camping, cooking, farm horses and animals Pets give company, pets are sweet, pets are around	a. Individual level /Interpersonal level/Role expectation/Socialization b. Individual level /community level/Role expectation/socialization c. Individual level /community level/Role strain relief/socialization d. Individual level /Interpersonal level/role strain relief/socialization

### Giving up on career goals and aspirations (individual level/societal/policy level/role expectations/division of labor)

Female FCgs often experience significant disruptions to their personal and professional aspirations due to the demands of their caregiving roles. For many female FCgs, the demands of caregiving require significant adjustments to employment, often resulting in reduced hours, declined opportunities, or complete withdrawal from the workforce. This was also affecting their career choices and opportunities while managing multiple caregiving and personal responsibilities. One of them shared:

*“I am the one who takes her to all her doctor’s appointments, coordinates grocery trips, and prepares supper. I also spend eight hours in the hospital daily. Because of these responsibilities, I now work part-time for an auction company doing their online listings.” (FCgs-4, 66 years).*

Some caregivers find themselves in unexpected situations despite their education and professional qualifications, highlighting the profound career sacrifices that caregiving can demand.

*“I have a small part-time job, but I never expected to be in this position—college-educated and a doctor, yet essentially a ‘broke doctor.’ It’s like an oxymoron, a bittersweet contradiction, like ‘jumbo shrimp.’ Even though I do not have money, I still feel incredibly blessed because I have my family, and that has not been taken from me.” (FCgs-1, 44 years).*

### Marital devotion (individual level/interpersonal level/gender roles/socialization/role expectation)

Most spousal caregivers perceived their caregiving roles as part of their marital commitment and obligation toward their partners. They pledged to sacrifice their own needs over their sick partners’ wishes. They reported a strong sense of commitment and devotion in their caring roles and found it to be meaningful and rewarding. One of the spousal caregivers shared as:

*“Yes, like I said, I was committed to the marriage. I said till death do us part and I meant it, so I stuck to the promise even though he was sick. …. I loved him I mean, he was my husband of 45 years, so I loved him, and I was willing to put up with it for that reason.” (FCgs-2, 72 years).*

Caregivers struggled to draw a fine line between maintaining their own well-being and respecting the autonomy and choices of the older adults in multiple instances during the caregiving process. This delicate balance often led to moments of grief and loss as well as experiencing compassion and fatigue.

### Subtheme 5: lack of social services and support

Female FCgs reported limited access to social support and services for caregivers in the rural areas. They had to struggle to find a paid caregiver for additional help. This further added to their caregiving burden and lack of *self-care*. One of them shared:

*“My dad falls quite often. Sometimes. I’m not sure I could get him up on my own since I am here alone. So, I called for my sister, sometimes she’s gone out of town. Then, I may have to call somebody call my pastor or other to come and help me, you know. If you are in a rural area, you do not know whom to call.” (FCgs-13, 58 years).*

### Caregivers’ coping and self-care strategies

Female FCgs were also asked about their self-care preferences and coping strategies that they often used to overcome their day-to-day caregiving stressors. Commonly reported strategies are provided below:

### Use of social media (individual level/interpersonal level/role expectation/socialization)

The use of social media was found to be a commonly used coping strategy to maintain socialization and engagement with friends and family. Regular emails, text messages, Facebook posts, virtual meetings using zoom or Team platforms, and online meditation apps were found to be useful coping and stress-management strategies. These strategies promoted their social and mental health and well-being. As one of them reported as:

*“I emailed relatives, and it was a big source of support for me because like I said, he (my spouse) would not read those emails. So, I could say whatever I felt like saying I did not have to put it through a filter…. I kept in touch with my children through email and they would email me back, which was helpful.” (FCgs-2, 72 years).*

*“I have an older and a younger sister who is very emotionally supportive. Sometimes I just send a text on my phone to thank them for their support and to express my feelings. They reply with messages like, ‘I’m so sorry you are going through this,’ and that’s enough for me, you know.” (FCgs-15, 59 years).*

Social media was also found to be a useful resource for learning about caregiving skills and enhanced health literacy. Female FCgs appreciated joining social media groups, watching online videos, and virtual tours to learn about caregiving skills*. “I belong to a few Zoom groups that I meet with regularly, which provides a nice social outlet.” Another added as: “taking that virtual tour exercise gave me insight into what it’s like for someone living with Alzheimer’s and dementia, which helped prepare me for providing care.*

### Online church meetings and meditation (individual level/community level/role expectation/socialization)

For some female FCgs, online church meetings and meditation apps helped them to minimize moral and spiritual distress and enhance peace and satisfaction. “*I use a meditation app online. I do not attend any classes; I just use the app to meditate when I am stressed. Another one added:* “*I cannot go to church, but our church posts services on YouTube. So, I can watch them when I am free by just connecting to the internet. This gives me a sense of peace and internal satisfaction.”*

### Participation in voluntary services (individual level /community level/role strain relief/socialization)

Voluntary services such as serving in Church and Bible school and involvement in physical *self-care* activities such as walking, gardening, cooking, reading books, watching movies, and bowling etc. were also helpful in providing some respite to caregivers. Engaging in these activities promoted a sense of belonging, provided distraction, and prevented social isolation.

*“Yes, I exercise to help me forget my worries. I’m an avid walker, and we have a hobby farm with horses and donkeys. It’s common for me to walk through the pasture and back to take care of them, which involves heavy lifting and some scooping. So, I try to stay active.” (FCgs-4, 66 years).*

Massage and chiropractic treatments were also reported by the female FCgs as another stress-management strategies. *“I found massage and chiropractic care effective for me. These treatments have significantly helped release the tension and stress in my body.”* (FCgs-15, 59 years).

### Owning a pet (individual level/interpersonal level/role strain relief/socialization)

For several rural caregivers owning a pet was reported as a vital source of companionship, something they genuinely enjoyed. As one of them suggested, “*To enhance your self-care, get a pet because you may not always have people around. Get a dog. I recommend the Havanese breed; they are very sweet. (FCgs-6, 73 years).* Caregivers also found pets as stress relievers and emotionally engaging. “*My best stress reducers are my pets. They give me company and keep me physically and emotionally alive.” (FCgs-4, 66 years).*

## Discussion

Our findings illuminate the self-care needs and preferences of rural female FCgs providing care for older family members in home or institutional settings. We analyzed these experiences through a gender equity and social lens. Our demographic findings align with previous caregiving studies showing most female FCgs are middle-aged (mean age 58 years) and 70% of them are married. About 66% provided care for their parents or in-laws at home for more than 6 years. About half of the caregivers were providing care for more than 20 h/week (37). These findings not only demonstrate the complexity and intensity of the caregiving burden taken up by the female caregivers but also points out to the gender roles and inequities, societal/cultural expectations, and available support systems within a rural context (38, 39).

Our findings also demonstrate the gender roles and expectations female caregivers’ experiences caring for their older family members. While managing multiple caregiving and personal responsibilities, they not only sacrifice their personal lives but also professional careers and aspirations. Notably, most caregivers (70%) reported higher levels of stress, i.e., 8 on a scale of 1–10 10 being the highest, underscoring the intense pressure they experience while caring for their older family members with chronic illness or disability. All these factors carry a profound impact on their personal agency, interpersonal relationships, behavioral coping and resilience also outlined in the socio-ecological resilience framework. Rurality further compounds these relationships and complexities given the lack of social and other support services. Given the heightened caregiving demands, these women caregivers are left with limited choices both in their personal and professional lives making them more vulnerable and at risk for poor caregiver resilience and wellbeing outcomes. Similar challenges have been reported in other caregiving studies, yet our findings extend these observations by highlighting the coping strategies used by rural female caregivers, including social media engagement, faith practices, and pet ownership indicating the urgent need to prioritize equitable gender-based caregiving policies and programs particularly in a rural context (39–43).

Our findings also point out that cultural norms and expectations can heavily influence how caregiving is perceived and performed. Most spousal caregivers view caregiving as their marital, moral and cultural responsibility often ending in high self-expectations and self-sacrifice (44, 45). Such behaviors are commonly observed among female FCgs who may feel a strong moral imperative to provide care for their loved ones rooted in strong sense of duty, family values, interdependence, and respect for elders. This strong sense of responsibility however may come at the expense of their own self-care and wellbeing and lead to caregiver burnout (46–49). Similarly, the social role theory also suggests that female caregivers are emotionally connected and more likely to sacrifice their social life while refraining from seeking help, even when support is available (29, 30). Recognizing these underlying values and motivations is therefore necessary to understand the caregiving concerns and preferences before designing policies and programs promoting female caregivers’ self-care and wellbeing.

Notably, rural female FCgs experienced shame, guilt, and feelings of helplessness when they are unable to fulfill their older adults’ expectations or care demands putting them at risk for increased anxiety and depression. They face communication barriers and harder transitions in care managing the physical and emotional needs of their caregivers and show signs of compassion fatigue (50). There is an increased likelihood that if such feelings not addressed can lead to unsafe and harmful behaviors and poor coping (48–50). Future studies are however needed to explain the phenomena of guilt and helplessness during the caregiving process and its consequences on the overall health and wellbeing of female FCgs in a rural context.

Our findings show that rural female FCGs increasingly use digital tools such as email, chat platforms, and Facebook—to stay socially connected, reduce loneliness, and manage the isolation common in rural settings. These technologies also improved caregivers’ health literacy by providing accessible information on treatment and care strategies, thereby strengthening confidence in their caregiving roles (51–54). Viewed through a resilience lens, digital innovations function as important supports at the individual and interpersonal levels, helping caregivers maintain coping resources despite geographic and service limitations. These findings highlight opportunities for rural health systems and community organizations to expand telehealth, virtual support groups, mobile health applications, and digital literacy initiatives tailored to caregivers. Policymakers can further enhance resilience by investing in rural broadband, funding virtual caregiving supports, and integrating technology into caregiver education and respite programs. Together, these strategies illustrate how digital interventions can mitigate rural isolation and bolster caregiver resilience across ecological levels.

Moreover, female FCgs also reported engaging in voluntary services and physical activities such as walking, gardening, cooking, and exercise to help them cope better with their caregiving roles and expectations. Similarly, faith and meditation were also reported as another coping strategy. Interestingly owning a pet was mentioned to overcome caregiver burnout. Pet was seen as a companion to enhance caregivers’ interpersonal, social, and emotional wellbeing (55). In summary, all these coping strategies helped them to promote their sense of belongingness and enhanced community participation (53–55). Future studies can consider evaluating these coping strategies in improving the *self-care* and wellbeing of female caregivers of older adults in a rural context.

These findings highlight the need for coordinated, gender-sensitive strategies that recognize the distinct realities of rural female caregivers (56, 57). Policies should move beyond one-size-fits-all approaches and instead support caregiver wellness through inclusive, value-based programs that respect cultural, faith, and family norms shaping caregiving roles (56, 57). Rural health systems and community-based organizations are well positioned to integrate technology-supported services, such as telehealth, virtual peer support, and digital education platforms, to reduce isolation and improve access to caregiving resources. Equally important is the expansion of affordable and accessible respite programs in rural communities to alleviate sustained caregiving strain and prevent burnout. From a resilience framework perspective, interventions should be designed to strengthen caregivers’ adaptive capacity across individual, interpersonal, and community levels. Finally, longitudinal research is needed to examine the long-term effects of caregiving on self-care practices, mental health, and wellbeing among rural female caregivers, providing evidence to guide sustainable policies and targeted interventions.

### Strengths and limitations

The study’s strength lies in its qualitative design which provided rich gender and context specific insights into the *self-care* needs and preferences of rural female FCgs of older adults. The in-depth interviews allowed female FCgs to share their personal experiences, offering valuable perspectives on the daily realities and complexities of caregiving, particularly in rural areas. Our findings also present several commonly used *self-care* practices among the rural caregivers. Our study limitations include its exclusive focus on female FCgs. Excluding male caregivers may have restricted additional insights into gender roles and inequities during the caregiving process. The small sample size drawn from a single Midwestern state also restricts the transferability of findings. Recruitment through community partners may have introduced selection bias, and the predominance of online interviews could have influenced female FCgs’ comfort in expressing emotionally sensitive content. Limited reporting on female FCgs’ race, and ethnicity status constrains interpretation of intersectional disparities. There is a likelihood that the self-reported data gathered through interviews may have introduced recall or social desirability bias. Another limitation of this study is the inclusion of caregivers providing care in both home and institutional settings. These two caregiving environments may present distinct challenges potentially leading to variations in caregiving experiences.

### Conclusion and future research recommendation

This study offers a thoughtful and grounded understanding of the daily realities faced by rural female caregivers, bringing forward their perspectives and highlighting the complexity of caregiving in geographically isolated settings. Drawing on socioecological resilience and social role theories, the findings point to an urgent need for supports that are sensitive to gendered caregiving expectations and responsive to the unique constraints of rural environments. Health and social care policies should incorporate wellness initiatives that are culturally attuned, faith-informed when appropriate, and designed to enhance caregivers’ ability to care for themselves as well as their loved ones. Digital tools such as telehealth platforms, virtual support groups, and mobile applications present promising avenues to reduce isolation and expand access to information and emotional support. Increased investment in rural respite services and technology-based interventions could further strengthen caregiver wellbeing and sustainability. Continued longitudinal research is needed to better understand the long-term effects of caregiving on rural women’s health and *self-care* practices, ultimately advancing public health, aging, and caregiver scholarship.

## Data Availability

The original contributions presented in the study are included in the article/Supplementary material, further inquiries can be directed to the corresponding author/s.
