# Supplementary material for: “It seems like a never-ending job”: voices of female caregivers of older adults in the rural communities
Source: Front Public Health. 2026 Jan 15;13:1751524. doi: 10.3389/fpubh.2025.1751524 (PMC12852003; doi:10.3389/fpubh.2025.1751524)
Supplement: Supplementary file 1 [file Supplementary_file_1.docx]

Appendix A

**Interview guide**

Tell me about yourself

Probes

Participant ID/Pseudonym: ____Age: __________ Place of Residence (Name of City/Town only):……….

Marital Status: Single Married Divorce/Separated Other

Sex/Gender: Male Female Other

Relationship to patient: Parent Spouse Sibling Friend Neighbor Other

Period of Caregiving: 3-6months, 6months to 1year 1-2years 3-5years 5-10year >than 10years

Income level Ranges (per anum): <20,000 20,000-40,000 40,000-60,000 >60,000

Unpaid caregiving hours/week

Diagnosis of Patient:

Are you (the caregiver) suffering from any illness? Yes/No If please specify! ……. Since how long …….

| **Questions** | **Probes** |
| --- | --- |
| **Challenges of Caregiving** | |
| What are some of the challenges you face as a family caregiver?    Do you think living in a rural area place you under any limitations/challenges or make a difference in your caregiving role?  Kindly describe your stress level on a scale of 0-10 where 0 is no stress and 10 is maximum stress | Physical-Diet, rest, sleep  Transportation/Visit to the facility  Travel to healthcare facility/Pharmacy, getting medications, managing care? Fall? Etc.  Support from healthcare providers?  Weather challenges |
| **Coping Strategies** | |
| What are the common coping mechanisms you use to promote your selfcare?  How much time you can spend for your own selfcare/wellness? | Hygiene  Hours of sleep  Nutrition  Exercise,  Do you attend any support group  Prayers/Meditation/Walk etc. |
| **Available Support system** | |
| What kind of support systems you have?  How helpful are they?  Do you need any help in household repair or similar things? How that help is provided? | Available supports-family, friends, neighbors, community systems  How helpful are these in your caregiving activities |
